# Dependency of codon usage on protein sequence patterns: a statistical study

**DOI:** 10.1186/1742-4682-11-2

**Published:** 2014-01-10

**Authors:** Mohammad-Hadi Foroughmand-Araabi, Bahram Goliaei, Kasra Alishahi, Mehdi Sadeghi

**Affiliations:** 1 Institute of Biochemistry and Biophysics, University of Tehran, Tehran, Iran; 2 Departement of Mathematical Sciences, Sharif University of Technology, Tehran, Iran; 3 National Institute of Genetic Engineering and Biotechnology (NIGEB), Tehran, 14155-6346, Iran

**Keywords:** Codon usage, Sequence analysis, Protein pattern, Pearson’s chi-squared test, Likelihood ratio test

## Abstract

**Background:**

Codon degeneracy and codon usage by organisms is an interesting and challenging problem. Researchers demonstrated the relation between codon usage and various functions or properties of genes and proteins, such as gene regulation, translation rate, translation efficiency, mRNA stability, splicing, and protein domains. Researchers usually represent segments of proteins responsible for specific functions or structures in a family of proteins as sequence patterns or motifs. We asked the question if organisms use the same codons in pattern segments as compared to the rest of the sequence.

**Methods:**

We used the likelihood ratio test, Pearson’s chi-squared test, and mutual information to compare these two codon usages.

**Results:**

We showed that codon usage, in segments of genes that code for a given pattern or motif in a group of proteins, varied from the rest of the gene. The codon usage in these segments was not random. Amino acids with larger number of codons used more specific codon ratios in these segments. We studied the number of amino acids in the pattern (pattern length). As patterns got longer, there was a slight decrease in the fraction of patterns with significant different codon usage in the pattern region as compared to codon usage in the gene region. We defined a measure of specificity of protein patterns, and studied its relation to the codon usage. The difference in the codon usage between pattern region and gene region, was less for the patterns with higher specificity.

**Conclusions:**

We provided a hypothesis that there are segments on genes that affect the codon usage and thus influence protein translation speed, and these regions are the regions that code protein pattern regions.

## Background

Researchers discovered the codon degeneracy phenomenon in 1965 [1]. The codon degeneracy is the phenomenon that more than one codon, codes for the same amino acid. Previous works studied the codon degeneracy phenomenon by different approaches such as correlation analysis and selective pressure evaluation [2,3]. They developed several measures to assess variability of codon usage among different conditions. For a summary of measures and their comparison please refer to the review by Suzuki et al. [4].

Previous researches show that different organisms use different ratios of synonymous codons [3]. Although, many researchers assign a unique codon usage to each organism, genes within the same organism use different ratios of codons [5,6]. This phenomenon may be due to some unknown biological roles of the codon usage [7].

Many researchers provided evidences in favor of codon selection in human [8]. They suggested different mechanisms for this selection, including translation efficiency [9-12], mRNA stability [13-15], and splicing control [12,16,17].

The literature suggests the codon usage as the translation efficiency controlling factor [8]. In the process of translation, the placement of tRNAs in the elongation phase is random. Also, the ribosome waits until a matching tRNA is correctly placed at its A site. Thus, translation of a gene which consists of codons with highly abundant tRNAs is fast. Through this mechanism, abundance of tRNAs along with codon usage affect the translation efficiency of genes [9,18-23].

These works show that living organisms use the relation between codon usage and tRNA abundance statically by choosing the codon usage and amount of tRNA isoacceptors in their genomes. Also, organisms use this relation dynamically by changing the amount of tRNAs in different conditions and tissues to control cell cycles [24-26]. For example, during amino acid starvation, organisms elevate the levels of tRNAs with high frequency in the genes that contribute to biosynthesis of amino acids [24,27].

Chamary et al. reported that some synonymous mutations, i.e. mutations that change the codon but not the resulting amino acid, affect the secondary structure of the mRNA [13]. This result is in favor of considering codon usage as a factor for mRNA stability. Also, as an example of the role of codon usage in the splicing control, researchers found synonymous mutations that disrupt the intron removal mechanism [16,28]. These findings, in addition to several other observations, confirm the codon selection phenomenon in organisms. For a complete survey on the codon selection in human refer to the review by Chamary et al. [8].

Recently, Najafabadi et al. claimed that, the codon usage, as a precoded factor, is involved in the gene regulation mechanism [29]. They support their claims by showing that co-regulated genes prefer similar codon usages. This could be considered as a breakthrough in the study of codon usage.

Sequence patterns or motifs represent conserved parts of protein sequences with common functional or structural features. They represent important sites such as enzyme catalytic sites, ligand binding sites, disulphide bonds, and many others [30].

In this paper, we investigated the relation between the codon usage and sequence patterns in related proteins. To the best of our knowledge, this paper is the first work reporting this relation. After confirming this relation, we suggested some underlying mechanisms causing this dependency.

We extracted sequence patterns from two popular protein pattern databases, namely, PROSITE [30] and Pfam [31]. We only used patterns which were in common in both databases. Experts have collected both these databases manually from results of experiments, and then enhanced them by computational tools. PROSITE database contains two types of entries: patterns and profiles. Patterns and profiles are both representing conserved residues, however, patterns represent conserved sites as regular expressions and profiles represent conserved domains as position specific matrices (PSM). “As these residues often are the more relevant for the biological function of the protein family or domain, further research can concentrate on them”. On the other hand, “profiles covering complete domains are more suitable for predicting protein structural properties” [30]. According to these facts, we used the pattern entities from the PROSITE database.

To develop PROSITE patterns, they first study reviews on families of proteins. Then, field experts extract biologically significant sites of families from literature. Finally, they produce a small pattern from alignment of significant sites. Thus, although PROSITE patterns are not biological in nature, they represent biological functions with high selectivity and specificity.

## Materials and methods

### Dataset

We selected protein patterns in common in two major pattern and family databases PROSITE [30] and Pfam [31]. Then, for each PROSITE pattern, we took proteins which were members of this pattern, and then we selected the genes that code these proteins from the EMBL-EBI database. We did not consider patterns with unknown positions, i.e. positions with ‘U’, and positions indicating start and end of proteins, i.e. positions with ‘ <’ and ‘ >’.

### Codon usage nomenclature

For a PROSITE pattern *p*, we defined **“pattern genes”** as the genes that encode positive hits of that pattern, i.e. proteins that positively match to the pattern. Based on “pattern genes” of a pattern *p*, **“pattern gene codon usage (GCU)”**${\text{gcu}}_{p}^{a}[\;c]$ represents the occurrence frequency of codon *c* for amino acid *a* in the “pattern genes” of the pattern *p*. Thus, ${\text{gcu}}_{p}^{a}[\;c]\;=n_{p}[\;c]/\underset{c^{\prime }}{\sum }n_{p}[\;c^{\prime }]$, where *n*_*p*_[ *c*] is the number of occurrences of codon *c* in “pattern genes” of pattern *p*, and the summation is over all codons that code for amino acid *a*. Note that, we considered the reading frames, and only count triplets that encode the amino acids.

We defined **“pattern region”** for a pattern *p*, as the subsequences of “pattern genes” that encode the subsequences of all proteins matching to the pattern *p*. Accordingly, we defined the **“pattern region codon usage (RCU)”**${\text{rcu}}_{p}^{a}[\;c]$ as the occurrence frequency of codon *c* corresponding to amino acid *a* in “pattern regions” of the pattern *p*. Thus, ${\text{rcu}}_{p}^{a}[\;c]\;=n_{p}^{\prime }[\;c]/\underset{c^{\prime }}{\sum }n_{p}^{\prime }[\;c^{\prime }]$, where $n_{p}^{\prime }[\;c]$ is the number of occurrences of codon *c* in “pattern regions” of the pattern *p*, and the summation is over all codons that code for amino acid *a*.

**Figure 1:**
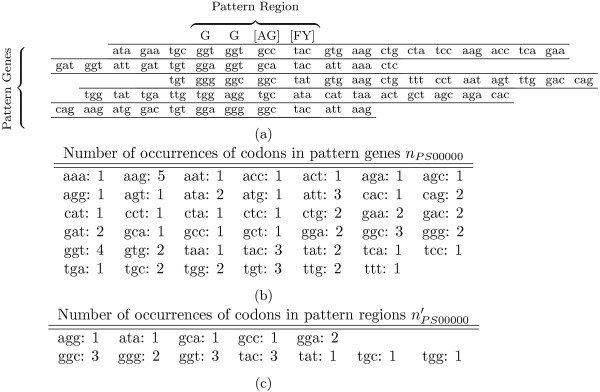
**Example of a PROSITE pattern and its corresponding pattern genes and pattern regions.(a)** The pattern contains four positions: “G”, “G”, “[AG]”, and “[FY]” and has five positive hits. Their corresponding genes making rows. Number of occurrences of codons **(b)** in pattern genes and **(c)** in pattern regions are also presented.

We present an example of a pattern, its “pattern genes”, and its “pattern regions” in Figure 1. Also this figure shows number of occurrences of codons in pattern genes and pattern regions.

### Statistical analysis

#### Test of equality of two codon usages

We tested the null hypothesis that two codon usages for a specific amino acid are equal, i.e. *A*^*a*^[ *c*] = *B*^*a*^[ *c*], where *A* and *B* are two codon usages and *A*^*a*^[ *c*] and *B*^*a*^[ *c*] are the occurrence frequency of codon *c* for amino acid *a* in codon usage *A* and *B*, respectively. In order to test it, we used the likelihood ratio test. More specifically we tested the equality of GCU (${\text{gcu}}_{p}^{a}[\;c]$) and RCU (${\text{rcu}}_{p}^{a}[\;c]$), by this method.

The test statistics *D* of the likelihood ratio test has, asymptotically, a chi-squared distribution and is given by *D* = -2 log(LNULL/LALTER), where LNULL is likelihood for the null model and LALTER is likelihood for the alternative model. The null model is the model with equal frequencies in two codon usages, and in the alternative model each codon in each codon usage has its own frequency. If the amino acid *a* has *#a* different codons, then the degrees of freedom for the null and alternative models are equal to *#a*-1 and 2(*#a*-1); and the degree of freedom for the chi-distribution is *#a*-1. We rejected the null hypothesis of equality of two codon usages at a significance level of *%*1.

Since the codon usage is meaningless for amino acids with only one codon, we excluded these amino acids from our investigations. These amino acids are Methionine (M) and Tryptophan (W). We considered the equality test for amino acid *a* and pattern *p*, only if each codon of amino acid *a* appears at least once in the pattern region, i.e. ${\text{rcu}}_{p}^{a}[\;c]\geq 1$ for all codons *c* which code for amino acid *a*, and also the amino acid *a* appears at least 30 times in the pattern region, i.e. $\underset{c}{\sum }{\text{rcu}}_{p}^{a}[\;c]\geq 30$ where the summation is over all codons *c* that code for amino acid *a*.

#### Test of randomness of RCU

We tested the randomness of RCU for pattern *p* and amino acid *a* with Pearson’s chi-squared test to test the null hypothesis of the equality of codon usage with a random theoretical background. Before testing the randomness, we normalized the bias which may be induced by distributional bias of pattern positive hits in the hierarchy of organisms. To remove this bias, we removed patterns which may cause evolutionary bias from the randomness test. Thus, we only considered patterns that their codon usage were extracted from evolutionary divergent organisms.

For each pattern *p*, we counted the number of occurrences of the pattern in each of the three main domains of life, namely, “Eukaryota” (*d*_*p*_[ *E*]), “Archaea” (*d*_*p*_[ *A*]), and “Bacteria” (*d*_*p*_[ *B*]). Then, we tested the randomness of *d*_*p*_, with test-statistics $X_{P}^{2}=\underset{k}{\sum }{\left(\;d_{p}[\;k]-D[\;k]\;\right)}^{2}/D[\;k]$, where the summation is over the three domains, and $D[\;k]=\underset{k}{\sum }d_{p}[\;k]/3$ is the random background. The degree of freedom for this test is 2. Patterns with p-values more than *%*5 are the patterns which we could not consider their distribution of positive hits as non-random.

Then, we tested the randomness of RCU for amino acid *a* and pattern *p*, if three conditions are satisfied. These three conditions are: 1) each codons of *a* appears at least once in the pattern region, 2) the amino acid appears at least 10 times in the pattern region, and 3) the randomness test of *d*_*p*_ was not rejected. The test-statistics $X_{R}^{2}$ for a RCU ${\text{rcu}}_{p}^{a}[\;c]$ and amino acid *a* is $X_{R}^{2}=\underset{c}{\sum }{\left(\;{\bar{n}}_{p}^{a}[\;c]-E[\;c]\;\right)}^{2}/E[\;c]$, where ${\bar{n}}_{p}^{a}[\;c]$ is the number of occurrences of codon *c* in the “pattern region” of the pattern *p*, and *E*[ *c*] is the theoretical background, and the summation is over all codons *c* that code for amino acid *a*. In this study, we used equal frequencies for the background codon usage, i.e. we let $E[\;c]=\left(\;,\underset{c}{\sum }{\bar{n}}_{p}^{a}[\;c],\;\right)/\mathrm{\#a}$, where *#a* is the number of codons that code for amino acid *a*, and the summation is over all codons *c* that code for amino acid *a*. The degree of freedom for this test is *#a*-1. We rejected the null hypothesis for p-values less than *%*1.

Note that, in our paper, for the test of equality of two codon usages we used the likelihood ratio test, however, for the test of randomness we used the Pearson’s chi-squared test. That is because in the test of equality of two codon usages, two codon usages represent observed values, while in the test of randomness, one of the codon usages, which is the background codon usage, is hypothetical. The likelihood ratio test is suitable for testing the equality of frequencies derived from observed values, while the Pearson’s chi-squared test is applicable for comparing observed and hypothetical frequencies.

In every statistical test, we considered p-values less than *%*1 to reject the null hypothesis. On the other hand, we considered results with p-values more than *%*5 as those for which we cannot reject the null hypothesis; for the randomness of distribution of pattern positive hits across domains we cannot reject the null hypothesis. Also, we used the limit of having at least 10 positive hits, and not having 30 positive hits, for occurrences in patterns for Pearson’s chi-squared test. That is because in the chi-squared test, the amount of available data is taken into account by the test.

### Mutual information

We considered GCU and RCU as random variables and evaluated their relative information by the mutual information measure. Let *t*_*p*,*o*_[ *c*] be the number of occurrences of codon *c* in organism *o* in “pattern genes” of the pattern *p*. Also, let $t_{p,o}^{\prime }[\;c^{\prime }]$ be the number of occurrences of codon *c*^′^ in organism *o* in “pattern regions” of pattern *p*. Let *t*_*p*_[ *c*,*c*^′^] be the number of simultaneous occurrences of codon *c* in pattern genes and codon *c*^′^ in pattern regions, i.e. $t_{p}[\;c,c^{\prime }]=\underset{o}{\sum }t_{p,o}[\;c]t_{p,o}^{\prime }[\;c^{\prime }]$. Now we can define *q*_*p*_[ *c*,*c*^′^] as the joint probability of occurrences of codon *c* in “pattern genes” and *c*^′^ in “pattern regions”, i.e. $q_{p}^{a}[\;c,c^{\prime }]\;=t_{p}[\;c,c^{\prime }]/\underset{c,c^{\prime }}{\sum }t_{p}[\;c,c^{\prime }]$, where the summation is over every possible pairs of codons that code for amino acid *a*.

The mutual information between GCU and RCU, for pattern *p* and amino acid *a* is

$$I_{p}^{a}=\underset{c,c^{\prime }}{\sum }q_{p}^{a}\left[\;,c,,,c^{\prime }\right]\log\left(\frac{q_{p}^{a}[\;c,c^{\prime }]}{q_{p}^{a}[\;c]{\bar{q}}_{p}^{a}[\;c^{\prime }]}\right)$$

where the summation is over every possible pairs of codons that code for amino acid *a*, and $q_{p}^{a}[\;c]$ and ${\bar{q}}_{p}^{a}[\;c^{\prime }]$ are marginal distributions of $q_{p}^{a}[\;c,c^{\prime }]$, which are $q_{p}^{a}[\;c]=\underset{c^{\prime }}{\sum }q_{p}^{a}[\;c,c^{\prime }]$ and ${\bar{q}}_{p}^{a}[\;c^{\prime }]=\underset{c}{\sum }q_{p}^{a}[\;c,c^{\prime }]$.

### Length and specificity of pattern

A PROSITE pattern *p* of size *n* is a sequence of sets of acceptable amino acids *A*_*i*_, for 1 ≤ *i* ≤ *n*. Also, each position *i*, may be repeated for a number of times which is either a number *α*_*i*_, or a range of possible values [ *β*_*i*_,*γ*_*i*_]. For each position *i* we defined a representative repeating number *r*_*i*_, which is *α*_*i*_ for positions with a certain repeating number, and (*β*_*i*_+*γ*_*i*_)/2 for positions with a range of possible repeating numbers. Accordingly, we define the representative length of a pattern *p* with size *n* as $L_{p}=\overset{n}{\underset{i=1}{\sum }}r_{i}$.

The specificity of a PROSITE pattern is the logarithm of the ratio of protein sequences that are matched to this pattern, to the number of all possible protein sequences with the same length. For a pattern *p*, the specificity *s*_*p*_ is defined as $s_{p}=-\underset{i}{\sum }r_{i}\log(|A_{i}|/20)$. For example a position that accepts any amino acid, i.e. an “x” position, does not increase the specificity of the pattern. Indeed, positions with more specific descriptions, i.e. positions with fewer acceptable amino acids, increase the specificity of a pattern more than highly degenerate positions.

We considered the relation between the representative length of patterns and our results. When considering the representative length of a PROSITE pattern, there is no difference between informative and highly degenerate positions. Thus, we provide the measure of specificity of patterns to take into account the amount of degeneracy of a pattern. We also considered the relation between the specificity of patterns and our results.

## Results

### Non-randomness of RCU

We filtered out patterns with non-random distribution of positive hits among domains of life. Among 320 patterns with more than 10 positive hits in three domains and at least one positive hit in each domain, for 61 patterns we could not reject the hypothesis that positive hits are distributed randomly among domains.

We tested the hypothesis of the randomness of RCU for 61 patterns and presented the results in Table 1. For each amino acid, we presented the total number of the cases, and the number of the cases for which the test rejected the hypothesis of the randomness of RCU.

**Table 1:** Percentages of the patterns for which the pattern codon usage is not random

**Amino acid**	**Number of codons for amino acid**	**Number of valid patterns**	**Number of patterns with non-random codon usage in pattern region**	**Percentages of patterns with non-random codon usage in pattern region**
C	2	24	15	%62.5
D	2	43	29	%67.4
E	2	41	25	%61.0
F	2	40	27	%67.5
H	2	33	16	%48.5
K	2	35	21	%60.0
N	2	38	15	%39.5
Q	2	38	22	%57.9
Y	2	37	15	%40.5
**I**	**3**	**52**	**48**	**%92.3**
**A**	**4**	**47**	**45**	**%95.7**
**G**	**4**	**54**	**46**	**%85.2**
**P**	**4**	**38**	**34**	**%89.5**
**T**	**4**	**48**	**42**	**%87.5**
**V**	**4**	**56**	**47**	**%83.9**
**L**	**6**	**50**	**49**	**%98.0**
**R**	**6**	**34**	**33**	**%97.1**
**S**	**6**	**40**	**39**	**%97.5**

We excluded amino acids with exactly one codon. If an amino acid appears 10 times in a pattern region, and each of its codons appears at least once in this region, we consider the pattern as a valid pattern for the amino acid. A pattern with non-random codon usage is a pattern for which the randomness hypothesis with respect to a completely random background is rejected.

Amino acids with low number of codons, i.e. 2, and 3, have random codon usage. While, amino acids with 4 or more codons have non-random codon usage as we showed in Table 1. Further details of the randomness test is available as Additional file 1.

### Difference between GCU and RCU

We compared GCU and RCU and presented the percentage of patterns for which these two codon usages are different in Table 2. Also, for each amino acid, we presented the number of codons that code for that amino acid, the number of valid patterns for that amino acid, and the number of cases for which these two codon usages are statistically non-equal in this table. Amino acids, in this table, are sorted ascending according to the number of their codons.

**Table 2:** Percentages of the patterns with different “pattern gene codon usage” and “pattern region codon usage”

**Amino acid**	**Number of codons of amino acid**	**Number of valid patterns**	**Number of patterns with non-equal pattern region codon usage and pattern gene codon usage**	**Percentages of patterns with non-equal pattern region codon usage and pattern gene codon usage**
C	2	561	276	%49.2
D	2	809	410	%50.7
E	2	772	393	%50.9
F	2	761	402	%52.8
H	2	609	284	%46.6
K	2	764	367	%48.0
N	2	724	361	%49.9
Q	2	639	310	%48.5
Y	2	668	341	%51.0
**I**	**3**	**860**	**565**	**%65.7**
**A**	**4**	**871**	**693**	**%79.6**
**G**	**4**	**937**	**751**	**%80.1**
**P**	**4**	**688**	**526**	**%76.5**
**T**	**4**	**824**	**634**	**%76.9**
**V**	**4**	**894**	**708**	**%79.2**
**L**	**6**	**831**	**739**	**%88.9**
**R**	**6**	**650**	**570**	**%87.7**
**S**	**6**	**724**	**668**	**%92.3**

We excluded amino acids with exactly one codon. If an amino acid appears 30 times in a pattern region, and each of its codons appears at least once in this region, we consider the pattern as a valid pattern for the amino acid.

There were 13586 valid cases for all the amino acids in Table 2. We observed different GCU and RCU in more cases for the amino acids with larger number of codons. For example for the amino acid “C”, which has 2 codons, in %49.2 of the cases gene region and pattern region codon usages were different, while, for amino acid “S”, that has 6 codons, this percentage is %92.3. Further details of the codon usage comparison tests are available as Additional file 2.

### Pattern length and codon usage in patterns

**Figure 2:**
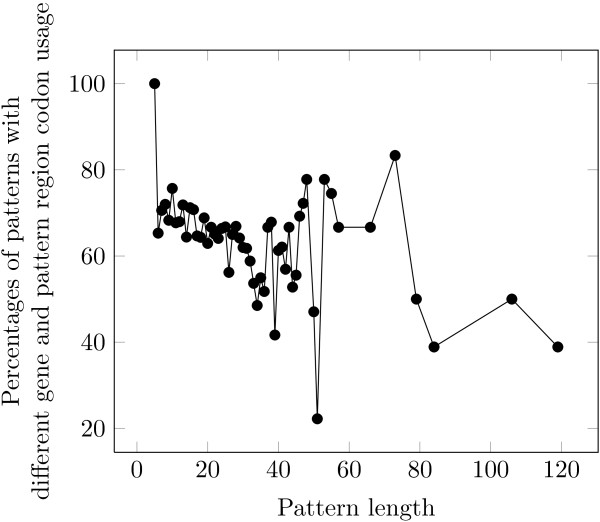
The effect of the pattern length on the percentages of the patterns which have different “pattern region codon usage (RCU)” and “pattern gene codon usage (GCU)”.

We investigated if there is any relation between the pattern representative length and the codon usage. Figure 2 shows the results. In order to produce this figure, for each representative length of PROSITE patterns, we gathered pairs of amino acids and patterns with the specified length. Then, we represented the percentages of these cases for which the hypothesis of equality of RCU and GCU is rejected.

As patterns get longer there is a slight decrease in the fraction of patterns with significant different RCU as compared to GCU. Low values of percentages for representative lengths more than 50 might be due to the small number of valid patterns observed at those lengths.

### Pattern specificity and codon usage

We analyzed the effect of pattern specificity (see the definition of pattern specificity) on the equality of RCU and GCU, and presented the results in Table 3.

**Table 3:** Percentages of the patterns for which the hypothesis of equality of “pattern region codon usage (RCU)” and “pattern gene codon usage (GCU)” is rejected, grouped by the specificity of the pattern

**Pattern specificity range**	**Number of valid patterns**	**Number of patterns with non-equal pattern region codon usage and pattern gene codon usage**	**Percentages of patterns with non-equal pattern region codon usage and pattern gene codon usage for amino acids**
[ 9-12)	12	10	%83.3
[ 12-15)	709	529	%74.6
[ 15-18)	2111	1420	%67.3
[ 18-21)	3792	2503	%66.0
[ 21-24)	3194	2112	%66.1
[ 24-27)	1516	992	%65.4
[ 27-30)	876	572	%65.3
[ 30-33)	444	282	%63.5
[ 33-36)	238	148	%62.2
[ 36-39)	184	130	%70.7
[ 39-42)	185	123	%66.5
[ 42-45)	128	69	%53.9
[ 45-48)	67	44	%65.7
[ 48-51)	57	32	%56.1
[ 51-54)	34	19	%55.9
[ 57-60)	21	7	%33.3
[ 60-63)	12	6	%50.0

The range of pattern specificities are divided to subranges of length 3, and the results are provided for each range of pattern specificities. If an amino acid appears 30 times in a pattern region, and each of its codons appears at least once in this region, we consider the pattern as a valid pattern for the amino acid. The “number of patterns with non-equal pattern region codon usage and pattern gene codon usage” is the number of cases for which the hypothesis of the equality of “pattern region codon usage (RCU)” and “pattern gene codon usage (GCU)” is rejected. The “Percentages of patterns with non-equal pattern region codon usage and pattern gene codon usage for amino acids” column represents the ratio of the number of cases with non-equal codon usages to the number of valid cases. We presented the rows with at least 10 valid patterns.

The difference is higher than %62 for rows with more than 150 valid patterns (Table 3). Also, the percentages of cases with different codon usages are decreasing, for the specificity ranges inside 9 to 36.

### Mutual information of GCU and RCU

We calculated the mutual information (MI) between GCU and RCU, and presented the results in Table 4. For each amino acid, we calculated how much information is in common between RCU and GCU. The value of 0 for the mutual information indicates total independence of the two codon usages, while, higher values of mutual information indicate more dependencies between these two codon usages. For each amino acid, we also presented percentage of the cases for which the mutual information is less than 0.0001 bits.

**Table 4:** Mutual information between “pattern gene codon usage (RCU)” and “pattern region codon usage (GCU)”

**Amino acid**	**Number of codons of amino acid**	**Number of valid patterns**	**Number of patterns with mutual information less than 0.0001 bits**	**Percentages of patterns with mutual information less than 0.0001 bits**	**Average of mutual information of the patterns**
C	2	561	491	%87.5	0.00009
D	2	809	735	%90.9	0.00007
E	2	772	693	%89.8	0.00005
F	2	761	686	%90.1	0.00007
H	2	609	525	%86.2	0.00007
K	2	764	689	%90.2	0.00006
N	2	724	660	%91.2	0.00005
Q	2	639	533	%83.4	0.00009
Y	2	668	600	%89.8	0.00007
I	3	860	715	%83.1	0.00008
A	4	871	646	%74.2	0.00015
G	4	937	659	%70.3	0.00018
P	4	688	454	%66.0	0.00023
T	4	824	601	%72.9	0.00016
V	4	894	683	%76.4	0.00012
L	6	831	499	%60.0	0.00026
R	6	650	355	%54.6	0.00035
S	6	724	491	%67.8	0.00017

The mutual information is computed between two random variables, namely, “pattern gene codon usage (GCU)”, and “pattern region codon usage (RCU)”. We excluded amino acids with exactly one codon. If an amino acid appears 30 times in a pattern region, and each of its codons appears at least once in this region, we consider the pattern as a valid pattern for the amino acid.

According to Table 4, we conclude that MI between GCU and RCU is very low for all the amino acids.

## Discussion

We studied the occurrence frequency of different codons of amino acids in two regions: in gene regions and in pattern regions. We used statistical tests to test the hypothesis of equality of these two codon usages. If, for a pattern, these two codon usages differ, we say that the pattern is discriminative.

The Pearson’s chi-squared test is a technique for testing the equality of an observed distribution with an expected distribution. Some previous works used the chi-squared test to show that the distribution of codons in various gene regions, such as regions corresponding to different secondary structures, is not random [32]. We used this technique to test the randomness of “pattern region codon usages (RCU)”. For each amino acid, we studied the codon usage of the amino acid in each pattern. We showed that the codon usage of amino acids in patterns are not random. It is more interesting that the genes corresponding to a pattern are from different organisms with different codon usages. This observation shows that there is a relation between pattern regions of the genes with similar patterns, even in different organisms.

The likelihood ratio test is a technique for testing the hypothesis of equality of two observed distributions. Researchers previously used the likelihood ratio test to compare the effect of translational speed and translational accuracy in the codon usage adaptation [33]. Here we used this technique to test the equality of codon usage of the genes (GCU) with the codon usage of the region of the genes that represent a pattern (RCU). We showed that in most of the cases the pattern regions use different ratio of codons, in comparison to gene regions. Of special interest is the proportional relation of the ratio of discriminative cases, and the number of codons for each amino acid. This discovery shows that whenever there are more freedom in choosing among codons, i.e. in amino acids with more codons, the organism uses different RCU and GCU in more cases. In fact, in this test, we showed in a more direct manner than the randomness test, that there is a relation between codon usages of pattern regions in different organisms.

Najafabadi et al. have shown that, in a specific organism, genes with different functions use different ratios of codon [29]. The results which we provided in the present paper is in agreement with the results of Najafabadi et al. Interestingly, our results show that the main factor behind the difference between the codon usage of genes with different functions is that they use different ratios of codons in their pattern regions (RCU).

We used the likelihood ratio test to test the effect of pattern length to the ratio of discriminative patterns. This test shows that shorter patterns are discriminative in most of the cases. In other words, patterns which may match to more sequences tend to be more discriminative. Note that, this relation is valid for patterns with length at most 50.

We defined another measure, which is called the specificity of pattern, to represent that how specific the description of a pattern is. As we described before, the specificity of a pattern represents the fraction of protein sequences with specific lengths that match to the pattern. Similar to the relation between the pattern length and the ratio of discriminative patterns, we observed the relation between the specificity of patterns and ratio of discriminative patterns. We showed that the patterns which match to more protein sequences, i.e. less specific patterns, use different pattern region (RCU) and pattern gene codon usages (GCU), in more cases.

Note that, in likelihood ratio tests, we used the p-value of %1. In this case, if the test fails, we should observe %1 positive results. Observation of more positive results, which we provided in this paper, shows that the tests are revealing a real world relation. In the randomness test we considered the dependency of codon usage to the organism and evolutionary distances between organisms, but we did not consider the evolutionary distance in other tests. That is because, in other tests, i.e. codon usage equality tests, we compared two codon usages, i.e. RCU and GCU, which are extracted from exactly the same set of organisms. However, in the randomness test, we compared a codon usage with a hypothetical background, thus, we considered the evolutionary distance between the set of selected organisms.

Mutual information of two random variables *X* and *Y* is a measure of independence of *X* from *Y*. Researchers have provided methods based on this concept to discriminate between coding and non-coding sequences [34]. Also, they have used mutual information to show the dependency of codon usage to the functions and expression levels of the genes [29]. We used this concept to evaluate the dependency of RCU and GCU. Our study of the equality of RCU and GCU, shows that for a pattern, there is a similarity in pattern regions, in different organisms. However, we showed by the mutual information that the dependency between RCU and GCU is very low. It means that, although we know that the organism tries to use a specific codon usage, however, organisms select the codon usage of pattern regions (RCU) independently.

This study, could be considered as an extension to the study of Najafabadi et al. [29]. They claimed that the codon usage is the mechanism which is used by organisms to regulate gene expression levels. They provided some theoretical and experimental tests to show this hypothesis. They chose some organisms, and for each, they examined the similarity of codon usages between co-expressed and co-function genes, i.e. genes with similar functions. They showed that the co-expressed and co-function genes use similar codon usages. They proposed their hypothesis about the genes within a specific organism. In contrast, with a different approach, we studied the relation between the codon usage of the protein members of motifs in different organisms. We showed that there is a universal, i.e. organism independent, tendency of using similar ratios of codon in pattern regions of proteins with similar patterns. Note that, protein sequence patterns represent preserved motifs in sequences which are due to a biological function.

Najafabadi et al. [29] showed that the similarity in codon usage between co-function proteins is higher than the codon usage similarity between two random proteins, and they did not analyze the origin of this difference in coding regions. Besides, we focused on the pattern regions of the proteins. From this point of view, our study explains the origin of differences in codon usages of different patterns, in coding region.

Najafabadi et al. provided an explanation for their findings that considers the effect of the codon usage in gene regulation. They proposed the codon usage as the factor which is used by organisms to control the expression of the genes. Indeed, they provided the mechanism that whenever an organism tries to activate a function, it changes the abundances of tRNAs, and consequently, some genes are switched on and off. However, this claim does not seem to be biologically valid. Indeed, the amount of available tRNAs may only affect the speed of translation, and consequently, the amount of proteins. In contrast, we provided a hypothesis that the organism uses this codon usage similarity between proteins, which participate in similar functions, to regulate protein expression levels, and does not use it to switch the genes on or off. Thus, the organism regulates the amount of available proteins by changing the amount of available tRNAs, and this mechanism is universal between organisms.

Why should pattern regions of a pattern in different organisms use similar codon usage? In fact, there should be a convergent evolution in codon usage of genes with a specific pattern, otherwise, mutations make these codon usages completely random. As researchers have claimed previously, codon usage is a factor for controlling protein expressions [29]. This claim is not only valid when considering genes individually, but also this is valid for a set of related proteins. In other words, the relative expression level of a set of proteins is controlled by the codon usage. This is the reason behind observing different codon usages in gene regions (GCU) and pattern regions (RCU).

## Conclusions

In this paper, we showed that the pattern region of the genes uses a non-random codon usage. Furthermore, we showed that the pattern region uses a different codon usage in comparison to the rest of the gene. Meanwhile, researchers have reported that the codon usage of the genes controls protein expression levels. Thus, the effect of pattern region on protein expression which is influenced by ratios of synonymous codons in this region, is not the same as the effect of the rest of the genes.

We observed more difference between codon usage in pattern region and gene region, for amino acids with more codes. On the other hand, for amino acids with more codes, there are more freedom in selection of the codon usage. Thus, we observed that, when there are more freedom in selection of the codon usage, the organism uses a more divergent pattern region codon usage, in comparison to the gene region. This mechanism for controlling protein expression levels, could be interpreted as an inter-organism mechanism for controlling protein expression levels.

## Competing interests

The authors declare that they have no competing interests.

## Authors’ contributions

All the authors contribute in designing statistical tests, analyzing the results, introducing the hypothesis, and preparing the manuscript. All authors read and approved the final manuscript.

## Supplementary Material

Additional file 1 **Randomness Test.** This file represents results of randomness test for different PROSITE patterns and different amino acids. In this test we statistically tested randomness of codon usage of pattern regions (RCU). Click here for file

Additional file 2 **Codon Usage Comparison.** This file represents results of comparison of codon usages of gene regions (GCU) and pattern regions (RCU) for different PROSITE patterns and different amino acids. This file also includes information about the patterns, such as representative length of the pattern, pattern specificity, mutual information of the pattern region codon usage, and the number of codons that codes for the amino acid. Click here for file
